# Effect of Initial Biomass Concentration on the Growth Kinetics of *Chlorella vulgaris* in Cylindrical Photobioreactors

**DOI:** 10.3390/bioengineering13070804

**Published:** 2026-07-13

**Authors:** Vadim A. Pavlov, Anatoly V. Grigorenko, Elizaveta M. Kovalenko, Marina E. Vavilkina, Mikhail S. Vlaskin

**Affiliations:** Joint Institute for High Temperatures of the Russian Academy of Sciences, 125412 Moscow, Russia; presley1@mail.ru (A.V.G.); lizloom@mail.ru (E.M.K.); vavilkina-marina@rambler.ru (M.E.V.)

**Keywords:** *Chlorella vulgaris*, microalgae, photobioreactors, initial biomass concentration, inoculum density, growth kinetics, lag phase, specific growth rate, Gompertz model, logistic model, model comparison

## Abstract

Microalgae of the genus *Chlorella* are widely used in biotechnology for biofuel production, wastewater treatment, and biomass generation. This study examined the effect of initial biomass concentration on the growth kinetics of *Chlorella vulgaris* cultivated in cylindrical photobioreactors. Experiments were performed in identical 4 L reactors under constant illumination (~300 µmol·m^−2^·s^−1^), aeration (0.5 vvm), and atmospheric CO_2_ (~0.04%). Four initial biomass concentrations (0.012, 0.053, 0.110, and 0.530 g·L^−1^) were tested in duplicate. Growth curves were fitted using the logistic, Gompertz, and Baranyi–Roberts models. The best-performing Gompertz model was further extended by relating its kinetic parameters to the initial biomass concentration, allowing biomass productivity to be evaluated as a continuous function of cultivation time and inoculum level. Initial biomass strongly affected growth dynamics. Increasing the initial concentration from 0.012 to 0.110 g·L^−1^ reduced the lag phase from 53.9 ± 5.1 h to 4.0 ± 6.9 h, while no distinct lag phase was observed at 0.530 g·L^−1^. Meanwhile, the maximum specific growth rate decreased from 0.0507 to 0.0251 h^−1^. The model-based analysis indicated that the optimal initial biomass concentration is time-dependent: higher values are preferable for short cultivations, whereas lower values become advantageous during prolonged cultivation. Although the predicted optimum partly lies between experimentally tested values and should be interpreted as exploratory rather than predictive, the proposed approach demonstrates the potential of model-assisted optimization for future process design, pending experimental validation.

## 1. Introduction

Microalgae have been considered in recent decades as a promising platform for a wide range of biotechnological applications, including biofuel production, feed additives and biologically active compounds, as well as wastewater treatment and CO_2_ utilization [1,2,3,4]. Among various species, *Chlorella vulgaris* has attracted particular attention due to its high growth rate, tolerance to environmental variations, and ability to produce significant amounts of biomass and accumulate valuable metabolites [1,5].

Large-scale cultivation of microalgae is carried out both in open systems (ponds and raceways) and in closed photobioreactors (PBRs), which provide a higher level of control over environmental parameters such as light intensity, temperature, and gas exchange [5,6]. In such systems, the key factors determining culture growth include light intensity, aeration rate, CO_2_ concentration, temperature, and nutrient availability [2,7,8].

In contrast to widely studied parameters such as aeration intensity, illumination, or CO_2_ concentration, the effect of the initial biomass concentration (inoculum density, X_0_) on microalgal growth kinetics has been studied to a much lesser extent. Nevertheless, this parameter plays a fundamental role in establishing the initial conditions of culture growth and can significantly affect the dynamics of all growth phases—from lag to stationary [9,10]. From a practical point of view, this parameter is important for inoculum preparation and for selecting the optimal initial biomass concentration.

On the one hand, increasing X_0_ leads to a reduction in the lag phase due to the fact that a larger fraction of cells is already in a physiologically active state and adapts more rapidly to environmental conditions [11]. On the other hand, high culture density enhances self-shading effects, reduces the efficiency of light utilization, and may limit CO_2_ mass transfer, which results in a decrease in the specific growth rate [12,13,14]. These effects are consistent with studies on the influence of culture density and illumination conditions on microalgal growth, where an increase in biomass concentration leads to enhanced self-shading and an earlier transition to the stationary phase [8,14,15,16].

Additionally, the effect of X_0_ is closely related to nonlinear light distribution within the photobioreactor. As biomass concentration increases, the light gradient becomes more pronounced, and a significant fraction of cells grows under light-limited conditions, resulting in reduced photosynthetic activity [14,15]. Similar conclusions have been reported in studies on microalgal growth modeling that account for light effects, where it has been shown that culture density directly affects local light availability and, consequently, growth kinetics [16].

Furthermore, the distribution of light within a photobioreactor is strongly influenced by its design, including geometry, diameter, and height-to-diameter ratio, as well as mixing and gas supply methods. These parameters determine the propagation of light within the culture volume and its penetration depth, which directly affects the photosynthetic activity of the cells. In particular, systems with a large optical path exhibit more pronounced self-shading effects, leading to the formation of light gradients and reduced efficiency of radiation utilization. The design features of photobioreactors also affect flow hydrodynamics and mixing regimes, which in turn determine the frequency of cell movement. Thus, reactor geometry and type are important input parameters in the analysis of growth kinetics and interpretation of experimental data [5,17].

Despite the existence of a number of studies addressing the influence of initial inoculum concentration on microalgal growth, a systematic analysis of this parameter in the context of kinetic models remains limited. In particular, it has been shown that changes in initial culture density can significantly affect both the maximum specific growth rate and biomass yield. For example, in studies of *Chlorella vulgaris* cultivation, a decrease in the initial concentration leads to an increase in the specific growth rate, whereas higher inoculum values are associated with a decrease due to reduced light penetration into the culture [18]. Similar trends have been reported in other studies: increasing the initial biomass concentration leads to reduced growth rates and productivity, which is attributed to self-shading effects and mass transfer limitations.

Moreover, the influence of initial concentration extends beyond growth kinetics to metabolic characteristics of the culture. In particular, it has been shown that the choice of inoculum size significantly affects productivity and lipid accumulation in two-stage cultivation processes of *Chlorella vulgaris*, where different X_0_ values lead to different growth trajectories. It has also been demonstrated that initial cell density affects photosynthetic activity and biomass accumulation dynamics at early growth stages.

Thus, although the influence of initial biomass concentration is recognized as an important factor, most studies consider it in an applied or technological context (e.g., productivity optimization, wastewater treatment), whereas the systematic relationship between X_0_ and the parameters of kinetic models (lag phase, μ_max_, carrying capacity) remains insufficiently explored. This determines the relevance of the present study.

To describe microalgal growth kinetics, sigmoidal models are widely used, including the logistic model, the Gompertz model, and the Baranyi–Roberts model [19,20,21]. These models allow quantitative evaluation of key growth parameters and are applied both for analysis of experimental data and for prediction of culture behavior under various conditions. At the same time, numerous comparative studies show that the Gompertz model often provides a more accurate description of experimental growth curves due to its greater flexibility in representing transitional regions [20,21,22].

At the same time, the parameters obtained from different models may differ significantly in their physical interpretability, especially with respect to the lag phase [19,23]. This makes it important to compare models not only in terms of fitting accuracy but also in terms of their ability to adequately represent biological processes.

Thus, despite the considerable number of studies devoted to modeling microalgal growth, the effect of initial biomass concentration as an independent control parameter remains insufficiently studied, particularly under atmospheric CO_2_ concentration and fixed aeration conditions [16]. It is important to note that in practical cultivation systems, factors such as pH, CO_2_ concentration, and light availability may vary and act as limiting factors. In the present study, cultures were grown under atmospheric CO_2_ without supplementation, and pH was not controlled, which may affect the interpretation of growth kinetics. These limitations are discussed in detail in the Section 4.

The aim of this study is to experimentally investigate the effect of the initial biomass concentration on the growth kinetics of *Chlorella vulgaris* in cylindrical photobioreactors and to select the mathematical model that most accurately approximates the experimental results.

To achieve this aim, the following objectives were formulated: to investigate growth dynamics at different values of X_0_; to determine the effect of initial biomass concentration on lag phase duration, specific growth rate, and biomass accumulation; to fit the experimental data using different kinetic models (logistic model with lag phase, Gompertz model, Baranyi–Roberts model); to perform a comparative analysis of the models in terms of fitting accuracy; and to evaluate the practical efficiency of different inoculation strategies for process optimization.

## 2. Materials and Methods

### 2.1. Experimental Setup and Cultivation Conditions

The effect of the initial biomass concentration on the growth of *Chlorella vulgaris* was investigated using a laboratory-scale system consisting of eight identical cylindrical photobioreactors (PBRs). Each reactor was a glass vessel with a total volume of 6 L (height 40 cm, diameter 14 cm) and a working volume of 4 L.

Mixing and gas supply were provided by continuous air sparging through diffusers installed at the bottom of each reactor. The aeration rate was maintained at 0.5 vvm for all experiments. The cultures were grown under atmospheric CO_2_ concentration (~0.04%) without additional carbon supplementation. No dissolved inorganic carbon (DIC) measurements or carbon balance calculations were performed during the experiments. Cultures were not axenic and were not sterilized prior to inoculation. Contamination was monitored by visual inspection and periodic microscopic examination throughout the cultivation period. No significant bacterial or fungal contamination was observed.

Illumination was provided by externally mounted white LED strips placed inside reflective enclosures surrounding each photobioreactor. Light intensity measured at the center of an empty reactor corresponded to approximately 300 µmol photons m^−2^ s^−1^ (≈18 kLux), with vertical variation ranging from 16 to 22.5 kLux. Measurements were performed using an LM-12 lux meter (JSC EKSIS, Moscow, Russia) with measurement uncertainty of ±10 lx.

Temperature inside the reactors was monitored every 4 h using contact thermometers Elemer (Moscow, Russia) with accuracy of ±1 °C. To compensate for evaporation losses, distilled water was periodically added, and all volume corrections were taken into account in subsequent biomass calculations.

Four initial biomass concentrations were tested. Each condition was performed in duplicate reactors to ensure reproducibility [Table 1].

**Figure 1:**
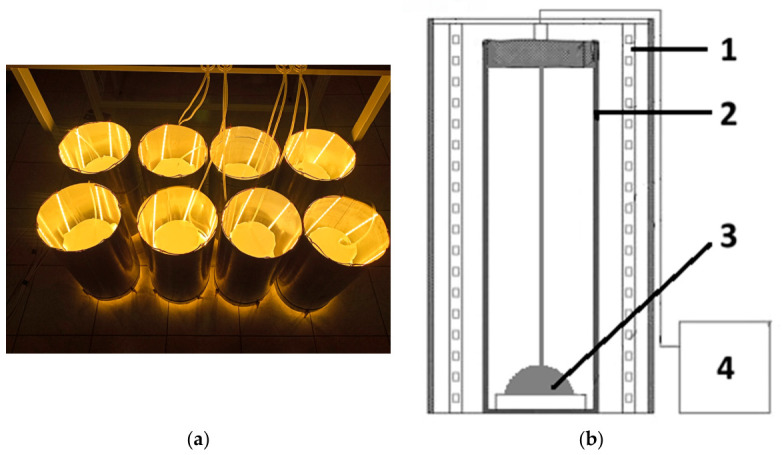
(**a**) Photo of the experimental setup (8-column PBR with 4 different initial biomass concentrations); (**b**) Schematic representation of a cylindrical PBR column. (1. LED strip—the light source providing photosynthesis; 2. glass vessel—the main reaction volume; 3. air sparger—an element for dispersing air in the liquid medium; 4. air compressor—supplies air for sparging).

A photograph of the experimental system and a schematic representation of the photobioreactor setup are provided in Figure 1. Details of the cultivation process are presented in Appendix A.

### 2.2. Cultivation Procedure

Batch cultivation of *Chlorella vulgaris* was carried out for 360 h. Inoculation was performed simultaneously in all eight photobioreactors to ensure identical initial conditions.

Sampling was conducted daily during the first 120 h of cultivation and subsequently at irregular intervals depending on the growth stage. All sampling and handling procedures followed a standardized protocol to minimize experimental variability. 

The collected data were recorded, tabulated, and used for subsequent kinetic analysis and model fitting.

### 2.3. Microalgal Strain and Growth Medium

The microalgal strain Chlorella vulgaris was obtained from the culture collection of the Faculty of Geography, Lomonosov Moscow State University (Moscow, Russia), and has been maintained in our laboratory collection without a formal deposit number in an international culture collection. The organism is a unicellular green microalga with spherical to slightly ellipsoidal cells ranging from 2 to 7 μm in diameter.

Prior to the experiments, the culture was acclimated to the selected cultivation conditions, including ambient CO_2_ concentration and room temperature. The inoculum was maintained under identical light, temperature, and nutrient conditions for several weeks to ensure physiological consistency at the time of inoculation.

A modified Tamiya medium was used for both inoculum preparation and experimental cultivation (pH ≈ 5.5). The medium contained (per liter of distilled water): KNO_3_ (5.0 g), KH_2_PO_4_ (1.25 g), MgSO_4_·7H_2_O (2.5 g), FeSO_4_·7H_2_O (0.009 g), EDTA (0.037 g), H_3_BO_3_ (2.86 mg), MnCl_2_·4H_2_O (1.81 mg), ZnSO_4_·7H_2_O (0.22 mg), (NH_4_)_6_Mo_7_O_24_·4H_2_O (0.018 mg), and NH_4_VO_3_ (0.023 mg). The initial pH of 5.5 was chosen to match the standard Tamiya medium composition without adjustment, as this represents typical conditions for batch cultivation without pH control. Although this pH value may be inhibitory for Chlorella vulgaris growth, it was maintained to simulate realistic cultivation conditions without active pH regulation.

The inoculum was directly transferred into the photobioreactors without prior concentration or washing, ensuring consistent initial conditions across all experiments. At the time of inoculation, the culture was in the late exponential growth phase with a biomass concentration of 0.5 g/L.

### 2.4. Analytical Methods

#### 2.4.1. Biomass Determination

Microalgal growth was monitored by measuring optical density at 750 nm (OD_750_) using a spectrophotometer SF-102 (Akvilon, Podolsk, Russia). When necessary, samples were diluted with distilled water to maintain OD values within the linear measurement range (0.3–0.6).

Biomass concentration was calculated using a calibration relationship:

(1) $$X=OD_{750}\cdot K\cdot n$$

where K is the calibration coefficient (0.4) based on previous work, and n is the dilution factor.

#### 2.4.2. Physicochemical Parameters

The pH of the culture medium was measured simultaneously with optical density using a pH meter pH-150MI (LLC Izmeritelnaya Tekhnika, Moscow, Russia) with accuracy of ±0.05). The initial pH of approximately 5.5 was not adjusted during cultivation to maintain consistent experimental conditions without active pH control. The pH dynamics observed during cultivation are discussed in detail in Section 3.1.

Temperature was recorded every 4 h using thermometers installed in each reactor (accuracy ± 0.5 °C). The readings were extracted from video recordings and logged for further analysis.

Evaporation losses were corrected by adding distilled water, and all biomass concentrations were adjusted to account for changes in the working volume.

### 2.5. Kinetic Models

#### 2.5.1. Logistic Model

The logistic model describes population growth under resource-limited conditions and is expressed as:

(2) $$\frac{dX}{dt}=\mu \cdot X\left(1-\frac{X}{K}\right)$$

where X is the biomass concentration (g·L^−1^), μ is the specific growth rate (h^−1^), and K is the carrying capacity (g·L^−1^).

The analytical solution of this equation is given by:

(3) $$X\left(t\right)=\frac{K}{1+\left(\frac{K-X_{0}}{X_{0}}\right)e^{-\mu t}}$$

This model captures sigmoidal growth behavior but does not explicitly account for the lag phase and may overestimate growth rates at low biomass concentrations.

#### 2.5.2. Logistic Model with Lag Phase

To incorporate the adaptation period, a lag phase parameter ($t_{\mathrm{lag}}$) can be introduced:

(4) $$X\left(t\right)=\frac{K}{1+\left(\frac{K-X_{0}}{X_{0}}\right)e^{-\mu \left(t-t_{\mathrm{lag}}\right)}},\;t\geq t_{\mathrm{lag}}\;$$

This modification allows a more realistic description of early-stage dynamics.

#### 2.5.3. Gompertz Model

The Gompertz model is widely used to describe asymmetric growth curves and is expressed as:

(5) $$X(t,X_{0})=X_{\max}exp\left(-exp\left(\frac{\mu_{\max}e}{X_{\max}}(\lambda -t)+1\right)\right)$$

where $X_{max}$ is the maximum biomass concentration, $\mu_{max}$ is the maximum specific growth rate, and λ is the lag-phase duration.

The model provides a flexible representation of growth deceleration and is particularly suitable when self-shading or adaptation effects are significant.

In contrast to the classical formulation, the model parameters were assumed to depend on the initial biomass concentration $X_{0}$. These dependencies were obtained by fitting empirical functions to the experimental data.

The maximum specific growth rate was approximated by a saturating function:

(6) $$\mu (X_{0})=\frac{\mu_{0}}{1+kX_{0}}$$

which reflects the decrease in effective growth rate at higher initial concentrations due to light limitation and self-shading.

The lag phase duration was approximated by a logarithmic function:

(7) $$\lambda (X_{0})=a\ln X_{0}+b$$

which captures the rapid variation in lag phase at low concentrations and weaker sensitivity at higher $X_{0}$.

Substituting these dependencies into the growth model yields a two-variable function X(t,$X_{0}$), which was used for further analysis.

The biomass gain was defined as:

(8) $$\Delta X(t,X_{0})=X(t,X_{0})-X_{0}$$

The volumetric productivity was defined as:

(9) $$P_{vol}(t,X_{0})=\frac{X(t,X_{0})-X_{0}}{t}$$

where X is the final biomass concentration (g·L^−1^), X_0_ is the initial biomass concentration (g·L^−1^), and Δt is the cultivation time (days). The units of volumetric productivity are g·L^−1^·day^−1^.

The optimal initial concentration was determined numerically as the value maximizing biomass gain over a discrete grid of $X_{0}$ values.

#### 2.5.4. Baranyi–Roberts Model

The Baranyi–Roberts model describes microbial growth with an explicit representation of physiological adaptation:

(10) $$y=\mu_{max}\cdot A\left(t\right)-\ln\left(1+\frac{\exp\left(\mu_{max}\cdot A\left(t\right)\right)-1}{\exp\left(C\right)}\right)$$

(11) $$A\left(t\right)=t+\frac{1}{\mu_{max}}\cdot \ln\left(\exp\left(-\mu_{max}t\right)+\exp\left(-\mu_{max}\lambda \right)-\exp\left[-\mu_{max}\left(t+\lambda \right)\right]\right)$$

where A(t) represents the adjustment function describing the transition from lag to exponential phase, and C is related to the maximum attainable biomass.

This model provides a smooth transition between growth phases and is particularly useful for describing cultures experiencing environmental stress or adaptation effects.

## 3. Results

### 3.1. Experimental Growth Dynamics

**Figure 2:**
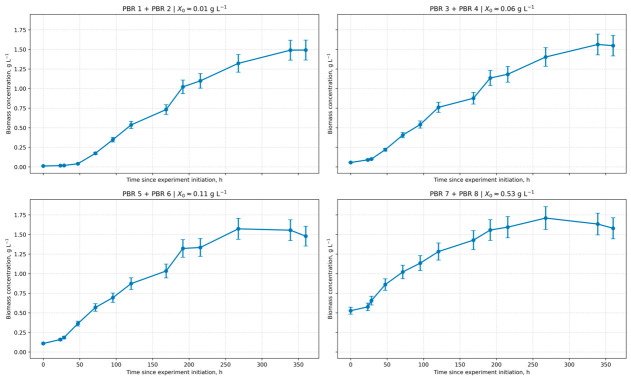
Biomass growth dynamics X(t) for PBRs at different initial concentrations X_0_. Error bars represent ±1σ.

Figure 2 shows the biomass growth dynamics of *Chlorella vulgaris* in photobioreactors at four different initial concentrations. Despite identical cultivation conditions (illumination, aeration, and medium composition), noticeable differences in growth kinetics are observed, which are attributed to differences in inoculum density. All growth curves exhibit a characteristic sigmoidal shape, including an initial growth phase, a phase of rapid biomass accumulation, and a subsequent stationary phase. However, the duration and intensity of these phases strongly depend on the initial biomass concentration.

At low initial concentrations (0.01–0.05 g·L^−1^), the culture requires more time to enter the exponential growth phase, but subsequently demonstrates a longer biomass accumulation period. In contrast, at higher initial concentrations (0.10–0.50 g·L^−1^), rapid growth is observed at early cultivation stages, while the transition to the stationary phase occurs earlier.

The maximum biomass concentration in all reactors is approximately 1.5–1.7 g·L^−1^, indicating the presence of a limiting carrying capacity under the given cultivation conditions.

**Figure 3:**
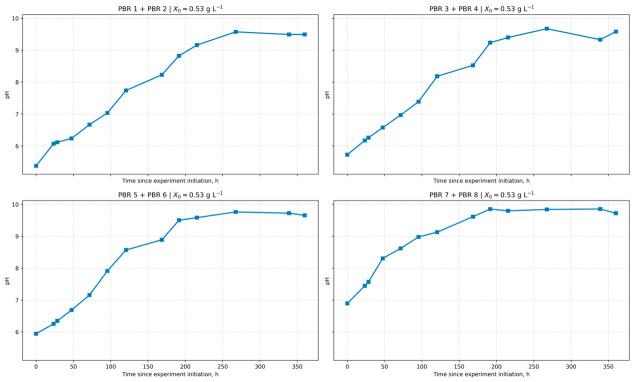
pH dynamics in PBRs at different initial concentrations X_0_.

During the experiment, a consistent increase in pH was observed in all photobioreactors, from initial values of 5.3–6.9 to 9.5–9.9 [Figure 3]. The most rapid increase was observed during the period of intensive biomass accumulation. The observed pH dynamics result from the equilibrium between CO_2_ consumption (which increases pH by removing carbonic acid) and nitrate assimilation (which consumes protons during reduction to ammonia). The initial pH of 5.5 was inhibitory, but the increase to near-neutral values during cultivation likely alleviated this limitation. Slightly higher initial pH values were observed at higher initial biomass concentrations; however, by the end of cultivation, pH values converged across all reactors.

### 3.2. Comparison of Growth Models

**Figure 4:**
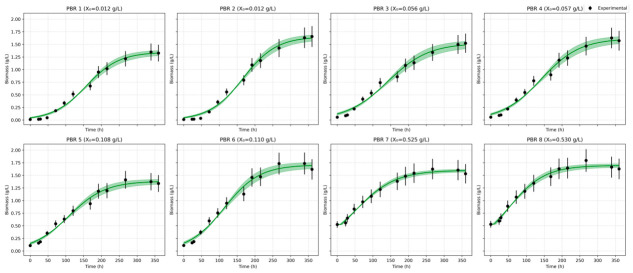
Logistic model with lag phase approximation. The green line is a mathematical model fit; the shaded band represents ±1σ.

**Figure 5:**
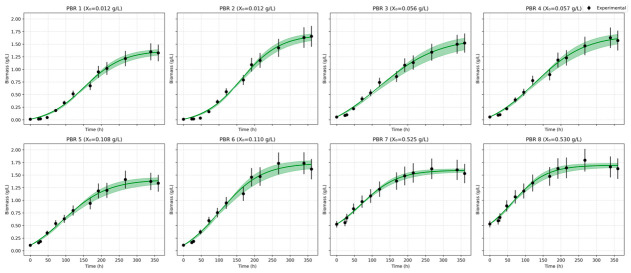
Baranyi–Roberts model approximation. The green line is a mathematical model fit; the shaded band represents ±1σ.

**Figure 6:**
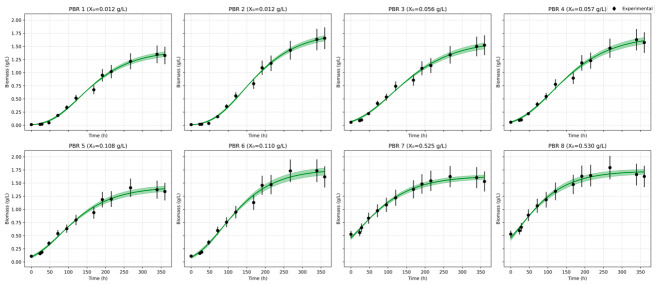
Gompertz model approximation. The green line is a mathematical model fit; the shaded band represents ±1σ.

To quantitatively describe growth kinetics, the experimental data were fitted using three commonly applied models: the logistic model with lag phase, the Gompertz model, and the Baranyi–Roberts model. The results of model fitting are presented in Figure 4, Figure 5 and Figure 6. The estimated parameters, including maximum biomass concentration, specific growth rate, and lag-phase duration, are summarized in Table 2, Table 3, Table 4 and Table 5.

Comparison of model performance showed that the Gompertz model provided the best agreement with experimental data. For most reactors, the coefficient of determination (R^2^) exceeded 0.99, while RMSE values were generally lower than those obtained with the other models.

According to the Gompertz model, the fitted lag-phase parameter decreased with increasing initial biomass concentration: from 53.9 ± 5.1 h at X_0_ = 0.012 g·L^−1^, to 17.9 ± 6.0 h at X_0_ = 0.056 g·L^−1^, to 4.0 ± 6.9 h at X_0_ = 0.110 g·L^−1^. At the highest initial concentration (X_0_ = 0.530 g·L^−1^), the fitted lag parameter became negative (−55.3 ± 9.0 h).

At the highest initial concentration (X_0_ = 0.530 g·L^−1^), the fitted lag parameter became negative (−55.3 ± 9.0 h), indicating that the model could not reliably resolve a distinct lag phase within the experimental time window. The negative lag phases observed at high inoculum densities may indicate that growth initiation occurred before the first measurement.

### 3.3. Specific Growth Rate Dynamics

**Figure 7:**
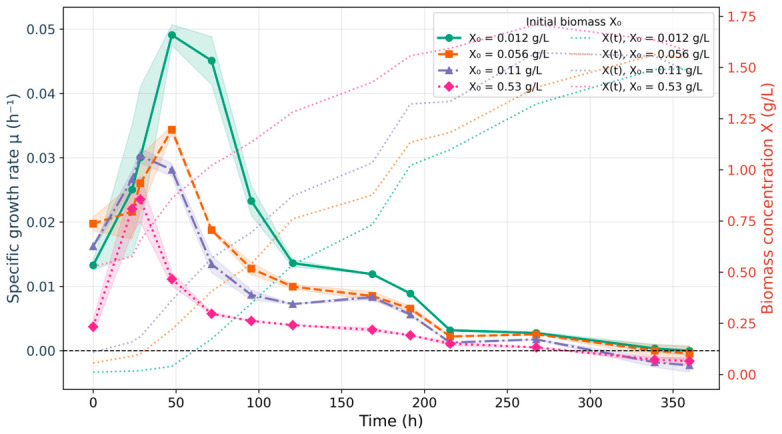
Time dependence of the specific growth rate μ(t) at different initial concentrations. Shaded band represents ±1σ.

The specific growth rate μ(t) was additionally calculated based on experimental data. Figure 7 presents the temporal evolution of μ for different initial biomass concentrations.

It is shown that lower initial concentrations lead to higher maximum specific growth rates, indicating more favorable conditions in terms of light availability and mass transfer. In contrast, at higher X_0_ values, a decrease in μ is observed, which may be attributed to self-shading effects and CO_2_ limitations.

A clear trend is observed: decreasing the initial biomass concentration results in an increase in the maximum specific growth rate $\mu_{max}$. Specifically, $\mu_{max}$ decreases from 0.0507 h^−1^ at X_0_ = 0.012 g·L^−1^ to 0.0360, 0.0319, and 0.0251 h^−1^ at X_0_ = 0.056, 0.11, and 0.53 g·L^−1^, respectively. Thus, within the studied range, $\mu_{max}$ doubles with decreasing X_0_.

The obtained values (0.025–0.051 h^−1^) are consistent with literature data for *Chlorella vulgaris*, where typical $\mu_{max}$ values range from 0.01 to 0.06 h^−1^. The observed dependence is also consistent with known effects of light limitation and self-shading: as biomass concentration increases, light availability per cell decreases, resulting in reduced growth rates. Additionally, under aeration with atmospheric air, CO_2_ mass transfer limitations may further contribute to the reduction of $\mu_{max}$ at high biomass concentrations.

### 3.4. Effect of Initial Concentration on Biomass Accumulation

**Figure 8:**
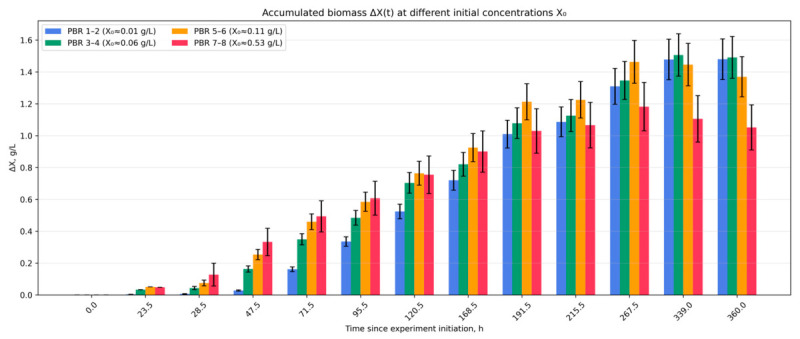
Accumulated biomass ΔX(t) at different initial concentrations.

The analysis of accumulated biomass (ΔX = X − X_0_) shows that reactors with higher initial concentrations do not exhibit a proportional increase in net biomass gain. Moreover, after a certain cultivation time (see Figure 8), systems with lower X_0_ begin to catch up with and even surpass those with higher X_0_ in terms of accumulated biomass.

**Figure 9:**
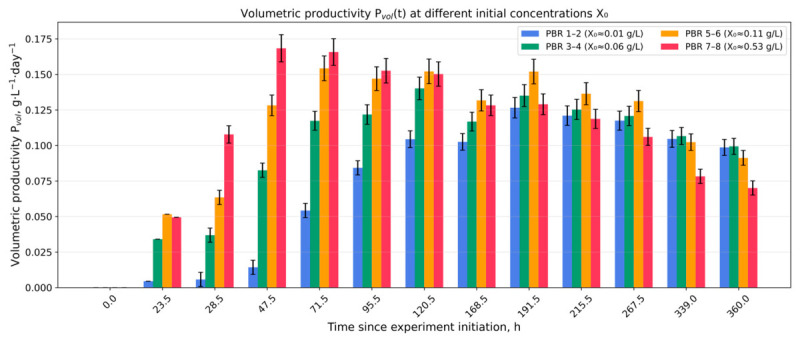
Volumetric productivity Pvol(t) at different initial concentrations.

In addition to the analysis of accumulated biomass, volumetric productivity Pvol was calculated to provide a complementary assessment of cultivation efficiency [Figure 9].

Gompertz model parameters also indicate that the optimal initial biomass concentration depends on cultivation time. At short times (up to ~160 h), the highest biomass gain is achieved at high X_0_ (~0.53 g·L^−1^), primarily due to the absence of a lag phase. At intermediate times (~160–280 h), medium initial concentrations (X_0_ ≈ 0.056–0.11 g·L^−1^) become more effective. For long cultivation periods (>280 h), the highest biomass accumulation is observed at low X_0_ (~0.012 g·L^−1^), which is explained by higher specific growth rates.

### 3.5. Model-Based Analysis of Optimal Initial Biomass Concentration

**Figure 10:**
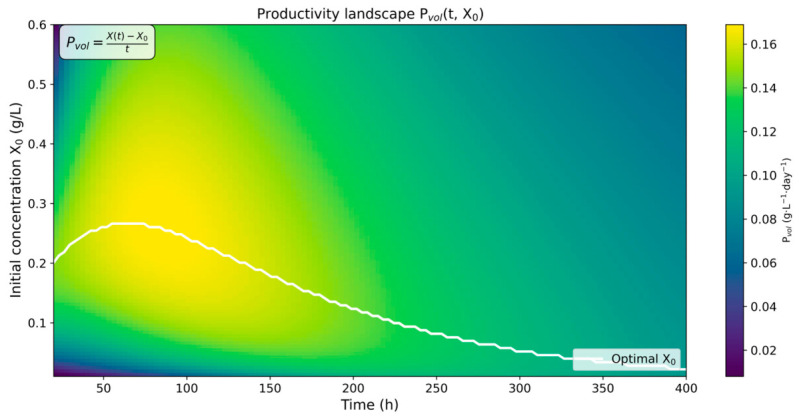
Productivity heatmap Pvol(t, X_0_).

The modified Gompertz model (5.2–5.4) was used to evaluate biomass productivity as a function of cultivation time and initial biomass concentration. Based on this approach, a continuous productivity landscape was constructed over the considered range of parameters. The resulting productivity map reveals a pronounced ridge corresponding to maximum biomass gain, indicating the existence of an optimal initial concentration that depends on cultivation time. This optimal trajectory was determined numerically by maximizing biomass gain at each time point over the considered range of initial concentrations. The heatmap representation provides an intuitive visualization of the productivity landscape and highlights the region of maximal biomass gain, supporting the interpretation of the optimal trajectory as a continuous feature rather than an artifact of discrete data points [Figure 10].

The results demonstrate that the optimal initial concentration is time-dependent: higher values are favorable at early stages (up to ~80 h) due to reduced lag phase, while lower values become optimal at longer cultivation times due to higher effective growth rates. It should be emphasized that the optimization results presented here are based on interpolation and partial extrapolation from four experimental data points. The predicted optimal region lies between experimentally studied concentrations; therefore, the heatmap (Figure 10) should be interpreted as exploratory rather than predictive, and the predicted optimum is hypothetical. Experimental validation at the optimal conditions is required to confirm these findings.

During the experiment, temperature inside the photobioreactors remained relatively stable at 22 °C ± 1 °C, except for an initial increase from 17 °C to 20 °C during the first hours. This transient temperature change may contribute to the presence of a lag phase, as temperature adaptation can influence the physiological state of cells and the duration of the lag phase.

## 4. Discussion

The obtained results demonstrate that the initial biomass concentration strongly influences the growth kinetics of *Chlorella vulgaris* cultivated in photobioreactors. In particular, substantial differences were observed in lag-phase duration, specific growth rate, and biomass accumulation dynamics across the investigated range of inoculum densities.

Cultures with low initial biomass concentration exhibited a prolonged lag phase but subsequently reached higher specific growth rates. In contrast, high initial concentrations resulted in rapid onset of biomass accumulation but lower effective growth rates during subsequent cultivation stages. Similar trends have been reported previously for microalgae cultivated under photoautotrophic conditions and are commonly associated with light attenuation effects in dense suspensions.

The observed decrease in specific growth rate with increasing initial biomass concentration is likely associated with reduced light availability per unit biomass caused by self-shading effects. Within the investigated range, the experimentally determined maximum specific growth rate decreased from 0.0507 h^−1^ at X_0_ = 0.012 g·L^−1^ to 0.0251 h^−1^ at X_0_ = 0.53 g·L^−1^, corresponding to an approximately twofold reduction. In photobioreactors, increasing cell density reduces photon flux penetration into the culture volume, thereby decreasing the average light available to individual cells. Under atmospheric aeration conditions, inorganic carbon transfer may additionally contribute to growth limitation at high cell densities. The maximum specific growth rates observed in this study (0.025–0.051 h^−1^) are lower than values reported for Chlorella vulgaris and related species under optimized cultivation conditions. For instance, Lakaniemi et al. reported μmax values of 2.0 day^−1^ (0.083 h^−1^) for C. vulgaris cultivated in flat plate photobioreactors [24], while Manhaeghe et al. observed μmax of 1.16 day^−1^ (0.048 h^−1^) under controlled light and temperature conditions [25]. For the closely related species Chlorella sorokiniana, Cuaresma Franco et al. measured μmax of 0.27 h^−1^ under simulated extreme winter conditions [26]. The classical work by Sorokin and Krauss demonstrated that Chlorella can achieve doubling times as short as 1.5 h under optimal steady-state conditions, corresponding to μmax of approximately 0.46 h^−1^ [27]. The substantially lower values observed in the present study are consistent with atmospheric CO_2_ conditions without supplementation and the absence of pH control, confirming that carbon limitation and suboptimal pH were significant factors constraining growth kinetics. It should be noted that the discrepancy between experimental and fitted $\mu_{max}$ values (approximately one order of magnitude) arises from the different calculation methods: experimental μmax represents the maximum instantaneous rate during exponential phase, calculated as the derivative of the ln(X) curve, while fitted $\mu_{max}$ is a model parameter optimized over the entire growth curve, including lag and stationary phases.

The pH increase observed during cultivation results from the equilibrium between CO_2_ consumption (which increases pH by removing carbonic acid) and nitrate assimilation (which consumes protons during reduction to ammonia). As dissolved CO_2_ is removed from the medium, the carbonate equilibrium shifts toward lower proton concentration, resulting in alkalization of the culture medium. The initial pH of 5.5 was inhibitory for Chlorella vulgaris growth, but the increase to near-neutral values during cultivation likely alleviated this limitation. Final pH values converged to approximately 9.5–9.9 across all reactors, which may indicate similar physicochemical limitations at late cultivation stages. The increase in pH might have been produced also by the nitrate used for growth, which is converted into ammonia during assimilation. We acknowledge several important limitations of this study that affect the interpretation of the results. First, pH and CO_2_ concentration were not controlled during cultivation. The initial pH of 5.5 was inhibitory for *Chlorella vulgaris* growth, and CO_2_ availability likely became limiting during the exponential phase, as evidenced by the relatively low maximum biomass concentration (1.5–1.7 g·L^−1^) achieved despite sufficient nitrate (5 g·L^−1^) and light (300 µmol·m^−2^·s^−1^) availability. This suggests that CO_2_ transfer and/or mixing efficiency were the primary limiting factors, rather than initial biomass concentration alone. Second, no dissolved inorganic carbon measurements or carbon balance calculations were performed, which prevents quantitative assessment of carbon limitation. Future studies should implement pH control, CO_2_ supplementation, and comprehensive carbon mass balance analysis to isolate the effect of initial biomass concentration.

Comparison of mathematical models showed that the Gompertz model provided the best agreement with experimental data among the tested approaches. In addition to high R^2^ values (0.982–0.996) and low RMSE values (0.037–0.066), the Gompertz model yielded lag-phase estimates demonstrating a systematic dependence on initial biomass concentration. The lag phase decreased from 53.9 ± 5.1 h at X_0_ = 0.012 g·L^−1^ to 4.0 ± 6.9 h at X_0_ = 0.11 g·L^−1^. At X_0_ = 0.53 g·L^−1^, the model produced negative lag-phase values, indicating that active biomass growth had likely already started before the beginning of measurements.

It should be noted that the negative lag-phase values obtained at high inoculum density do not necessarily indicate the physical existence of a “negative lag phase”. Instead, they reflect the inability of empirical growth models to resolve a distinct adaptation stage under conditions where active biomass growth had already begun at the start of measurements. However, we acknowledge that this interpretation requires careful consideration of parameter identifiability. Comparison of constrained (λ ≥ 0) and unconstrained fits suggests that the negative values are within the confidence intervals of the model parameters, indicating that the growth dynamics at high X_0_ are characterized by immediate exponential phase onset. An additional aspect of this work is the model-based reconstruction of biomass productivity as a continuous function of cultivation time and initial biomass concentration. Unlike conventional analysis limited to discrete experimental conditions, the proposed framework enables interpolation between experimentally tested inoculum levels and allows visualization of the predicted productivity landscape. The resulting analysis suggests the existence of a time-dependent favorable range of initial biomass concentrations rather than a single universal optimum.

At short cultivation durations, higher initial concentrations are advantageous because they reduce or eliminate the apparent lag phase. However, at longer cultivation times, lower inoculum densities become more favorable due to higher effective growth rates. Additionally, we acknowledge that formal statistical analysis was not performed in this study due to the limited number of replicates (n = 2). Although the predicted optimum partly lies between experimentally studied concentrations and should be interpreted as exploratory rather than predictive, the obtained results demonstrate the potential of model-assisted optimization for cultivation planning and photobioreactor process design, pending experimental validation at the predicted optimal conditions.

The proposed approach remains semi-empirical and is subject to several limitations. In particular, the model does not explicitly account for spatial light distribution, photon absorption, or inorganic carbon transport inside the photobioreactor. Since light propagation in dense microalgal suspensions is highly complex and depends on both absorption and scattering phenomena, incorporation of radiative transfer effects may substantially improve predictive capability.

## 5. Conclusions

In the present study, the growth kinetics of *Chlorella vulgaris* cultivated in photobioreactors at different initial biomass concentrations were investigated using experimental measurements and mathematical modeling. The results demonstrated that the initial biomass concentration strongly affects lag-phase duration, specific growth rate, and biomass accumulation dynamics.

Low initial biomass concentrations resulted in prolonged lag phases but higher effective specific growth rates. According to the Gompertz model, the lag phase decreased from 53.9 ± 5.1 h at X_0_ = 0.012 g·L^−1^ to 4.0 ± 6.9 h at X_0_ = 0.11 g·L^−1^, while no distinct lag phase was detected at X_0_ = 0.53 g·L^−1^. At the same time, the experimentally determined maximum specific growth rate decreased from 0.0507 h^−1^ to 0.0251 h^−1^ with increasing initial biomass concentration.

Analysis of biomass accumulation demonstrated that the preferable inoculum density depends on cultivation duration. High initial biomass concentrations were advantageous at short cultivation times due to rapid onset of growth, whereas lower concentrations became more favorable during prolonged cultivation because of higher effective growth rates.

Among the tested approaches, the Gompertz model provided the best agreement with experimental data, with R^2^ values of 0.982–0.996 and RMSE values of 0.037–0.066. In addition, this model demonstrated the most physically interpretable dependence of kinetic parameters on inoculum density.

A model-based reconstruction of biomass productivity was further used to obtain a continuous productivity landscape as a function of cultivation time and initial biomass concentration. The proposed framework indicated the existence of a time-dependent favorable region of inoculum densities and enabled interpolation between experimentally tested conditions. Although the predicted optimum partly lies between experimentally studied concentrations, the obtained results demonstrate the potential of model-assisted optimization for cultivation planning and photobioreactor process design.

The proposed approach remains preliminary due to several limitations. First, the lack of pH and CO_2_ control means that the observed growth patterns reflect the combined effects of multiple limiting factors (initial biomass concentration, pH inhibition, CO_2_ limitation, and light attenuation) rather than X_0_ alone; consequently, the conclusion that initial biomass concentration was the primary factor controlling growth should be interpreted with caution. Second, the relatively low maximum biomass concentration (1.5–1.7 g·L^−1^) achieved despite sufficient nitrate and light indicates that CO_2_ availability and/or mixing efficiency were likely the primary limiting factors. Third, the limited number of experimentally tested concentrations, the absence of direct dry weight validation of biomass calibration, formal statistical analysis, and explicit radiative transfer modeling restrict the predictive capability of the current framework. Future studies should therefore implement pH control, CO_2_ supplementation, comprehensive carbon mass balance analysis, a finer range of inoculum densities, incorporation of light transfer effects into mechanistic growth models, and statistical validation to isolate and confirm the effect of initial biomass concentration on growth kinetics.

## Figures and Tables

**Table 1:** Initial biomasses in the PBRs.

PBR	Biomass Concentration, g/L
1–2	0.012
3–4	0.056
5–6	0.110
7–8	0.530

**Table 2:** Fitted kinetic parameters for the logistic model.

PBR	Initial Biomass Concentration (g/L)	K, g/L	±K, g/L	$\mu_{max}$, h^−1^	±$\mu_{max}$, h^−1^	R^2^	RMSE
1	0.012	1.286	0.053	0.030	0.001	0.9736	0.0825
2	0.012	1.575	0.063	0.030	0.001	0.9766	0.0946
3	0.056	1.436	0.063	0.024	0.001	0.9675	0.0942
4	0.056	1.543	0.060	0.024	0.001	0.9744	0.0906
5	0.110	1.352	0.041	0.023	0.001	0.9800	0.0667
6	0.110	1.669	0.051	0.024	0.001	0.9801	0.0841
7	0.530	1.621	0.030	0.016	0.001	0.9879	0.0434
8	0.530	1.715	0.035	0.017	0.001	0.9827	0.0568

**Table 3:** Fitted kinetic parameters for lag-logistic model.

PBR	Initial Biomass Concentration (g/L)	K, g/L	±K, g/L	$\mu_{max}$, h^−1^	±$\mu_{max}$, h^−1^	λ, h	±λ, h	R^2^	RMSE
1	0.012	1.345	0.044	0.022	0.002	−62	19	0.9897	0.0525
2	0.012	1.652	0.052	0.021	0.002	−65	19	0.9912	0.0579
3	0.056	1.522	0.062	0.017	0.002	−46	17	0.9846	0.0649
4	0.056	1.615	0.059	0.018	0.002	−39	15	0.9867	0.0653
5	0.110	1.383	0.044	0.019	0.002	−18	11	0.9848	0.0580
6	0.110	1.706	0.056	0.020	0.002	−19	12	0.9845	0.0738
7	0.530	1.600	0.026	0.018	0.002	10	4	0.9916	0.0563
8	0.530	1.696	0.033	0.019	0.002	9	6	0.9860	0.0512

**Table 4:** Fitted kinetic parameters for Gompertz model.

PBR	Initial Biomass Concentration (g/L)	$X_{max}$, g/L	±$X_{max}$, g/L	$\mu_{max}$, h^−1^	±$\mu_{max}$, h^−1^	λ, h	±λ, h	R^2^	RMSE
1	0.012	1.424	0.048	0.0067	0.0004	50	5	0.9943	0.038
2	0.012	1.673	0.052	0.0079	0.0004	57	5	0.9964	0.037
3	0.056	1.618	0.061	0.0063	0.0004	15	6	0.9930	0.044
4	0.056	1.711	0.063	0.0070	0.0004	20	6	0.9926	0.049
5	0.110	1.530	0.049	0.0068	0.0005	0	7	0.9882	0.051
6	0.110	1.761	0.064	0.0085	0.0007	8	7	0.9878	0.066
7	0.530	1.636	0.037	0.0075	0.0006	−60	9	0.9876	0.044
8	0.530	1.729	0.044	0.0089	0.0008	−51	9	0.9821	0.058

**Table 5:** Fitted kinetic parameters for Baranyi–Roberts model.

PBR	Initial Biomass Concentration (g/L)	$X_{max}$, g/L	±$X_{max}$, g/L	$\mu_{max}$, h^−1^	±$\mu_{max}$, h^−1^	λ, h	±λ, h	R^2^	RMSE
1	0.012	1.372	0.044	0.0185	0.0021	−100	29	0.9929	0.043
2	0.012	1.689	0.052	0.0183	0.0019	−105	28	0.9942	0.047
3	0.056	1.622	0.080	0.0119	0.0021	−146	41	0.9928	0.045
4	0.056	1.677	0.070	0.0141	0.0021	−103	38	0.9919	0.052
5	0.110	1.510	0.052	0.0159	0.0029	−56	33	0.9880	0.052
6	0.110	1.736	0.064	0.0169	0.0029	−52	31	0.9876	0.067
7	0.530	1.597	0.030	0.0192	0.0032	22	14	0.9899	0.040
8	0.530	1.696	0.038	0.0208	0.0043	19	17	0.9843	0.054

## Data Availability

No new data were created or analyzed in this study. Data sharing is not applicable to this article.

## Abbreviations

The following abbreviations are used in this manuscript:

PBR	PhotoBioReactor
RMSE	Root Mean Squared Error

## Appendix A

During the long-term cultivation of *Chlorella vulgaris* in open photobioreactors, it was found that aeration led to a noticeable evaporation of the culture medium. This resulted in a gradual decrease in the total liquid volume, which, in turn, caused a systematic overestimation of the measured biomass concentration.

To compensate for these volume losses, three refills of mineralized water were carried out during the experiment, and the added volumes were recorded. Thus, for the first three time intervals—from the initial volume to the first refill, from the first to the second, and from the second to the third refill—the culture volume was known (V_0_ to V_1_, V_0_ to V_2_, and V_0_ to V_3_, respectively).

After the third refill, direct volume measurements were no longer performed. To reconstruct the temporal profile of the culture volume during the subsequent stage, a linear evaporation model was applied. It was assumed that evaporation after the third refill occurred at an average rate similar to that determined during the preceding interval.

The culture volume during this period was estimated using the following relation:

(A1) $$V\left(t\right)=V_{0}-\nu \cdot \left(t-t_{3}\right),\;\;t>t_{3},$$

where V_0_ = 4000 mL is the culture volume immediately after the third refill, t3 is the time of the last refill, and ν is the mean evaporation rate determined experimentally.

The measured biomass concentrations were corrected for the changing culture volume as follows:

(A2) $$X_{\mathrm{corr}}\left(t\right)=X\left(t\right)\cdot \frac{V\left(t\right)}{V_{0}}$$

which allowed the removal of systematic overestimation in biomass concentration caused by the gradual evaporation of the culture medium during the final stage of cultivation.
